# Variability in Response to Valsartan and Its Relationship With *AGT M235T* Genotype and Other Nongenetic Parameters Among a Sample of Hypertensive Individuals in Jordan: A Prospective Pilot Study

**DOI:** 10.1002/hsr2.70611

**Published:** 2025-04-16

**Authors:** Hussein Alhawari, Yazun Jarrar, Malek Zihlif, Ayman Wahbeh, Sameeha Alshelleh, Khaled Ojjoh, Dalia Abdelrazaq, Hussam Alhawari

**Affiliations:** ^1^ Department of Internal Medicine School of Medicine, The University of Jordan Amman Jordan; ^2^ Department of Basic Medical Sciences Faculty of Medicine, Al‐Balqa Applied University Al‐Salt Jordan; ^3^ Department of Pharmacology School of Medicine, The University of Jordan Amman Jordan

**Keywords:** AGT M235T genotype, hypertension, interindividual variation, personalized medicine, valsartan

## Abstract

**Background:**

Valsartan, an angiotensin receptor antagonist widely used in hypertension and heart failure management, exhibits noticeable interindividual variation in response among hypertensive patients at the University of Jordan Hospital. The *angiotensinogen (AGT)* gene variant *M235T*, a functional genetic variant, influences the renin‐angiotensin system.

**Aims:**

This study aims to explore interindividual variations in the valsartan response, considering genetics, particularly the *AGT M235T* variant, and other nongenetic factors.

**Methods:**

This cohort study involved 95 unrelated Arabic Jordanians diagnosed with essential hypertension. Systolic (SBP) and diastolic blood pressure (DBP) measurements were taken at the initiation of 160 mg valsartan and after 1 month of treatment, assessing the valsartan response for each patient. Genetic analysis of *AGT M235T* was done using the polymerase chain reaction‐restriction fragment length polymorphism genotyping method. Anthropometric data were collected from University of Jordan Hospital computer records.

**Results:**

Valsartan response assessment revealed diverse individual responses, the response to valsartan varied, with SBP reductions from < 10 to > 70 mmHg and DBP from < 2 to 30 mmHg. Patients with homozygous *AGT M235T* genotypes showed a less significant response (*p* < 0.05) to valsartan than heterozygous and reference genotypes. Additionally, results indicated a positive correlation of age (*p* = 0.03) and a negative correlation of height (*p* = 0.02–0.04) with the valsartan response. Regression analysis demonstrated that the patients' sex significantly influenced the valsartan response (*p* < 0.05).

**Conclusions:**

This study identifies the *AGT M235T* genotype as a potential genetic contributor to variability in the valsartan response. Associations with age, height, and sex underscore the importance of considering genetic and demographic factors in tailoring valsartan therapy, for advancing personalized hypertension management.

## 1 Introduction

Hypertension is identified when systolic readings exceed 140 mmHg and/or diastolic readings are over 90 mmHg [1]. The prevalence of hypertension is high among the world [2]. In Jordan, recent research indicates a hypertension prevalence of 41.4% in men and 28.3% in women, suggesting an increasing trend in hypertension cases and a subsequent rise in coronary heart disease risk [3].

The renin‐angiotensin system (RAS) plays a crucial role in regulating blood pressure, fluid balance, and electrolyte levels. Within this system, angiotensinogen (AGT), produced by the liver, is a vital component [4]. Research has particularly focused on the *AGT* gene for its association with cardiovascular diseases [5].

Angiotensin receptor antagonists, also known as Angiotensin II Receptor Blockers (ARBs), are primarily used in the management of hypertension and heart failure. They work by selectively blocking the binding of angiotensin II, a potent vasoconstrictor, to its receptor sites in the vascular smooth muscle and adrenal gland. This action prevents angiotensin II from exerting its effects, which include vasoconstriction, aldosterone secretion, and stimulation of water and sodium reabsorption by the kidneys, leading to lower blood pressure, reduced blood volume, and, consequently, decreased workload on the heart [6]. ARBs are particularly beneficial for patients who experience side effects from ACE inhibitors, another class of cardiovascular medication, as they offer a similar therapeutic effect without the cough associated with ACE inhibitors [7]. Additionally, ARBs are used in patients with chronic kidney disease, as they have been shown to slow the progression of renal deterioration [8].

There is an interindividual variation in the response to ARBs. This interindividual variation can be due to the genetic and nongenetic factors [9]. It was found that the *angiotensin II type 1 receptor* gene A1166C variant influenced the response toward ARBs [10].

The *angiotensinogen (AGT*) gene is located on chromosome 1q42‐43. This gene is highly polymorphic. One of the major functional *AGT* variants is the non‐synonymous M235T variant. This genetic variant involves a substitution of thymine to cytosine at nucleotide 704 (T704C), leading to an amino acid substitution from methionine (M) to threonine (T) at position 235 [5].

There is a lack of studies regarding the influence of *AGT M235T* genetic variant on the response of ARBs among hypertensive patients in Jordanians. Therefore, this study aims to investigate whether the *AGT M235T* genetic variant and other nongenetic anthropometric parameters, such as age, weight, and gender, affect the response to valsartan in Jordanian hypertensive patients. It examines if there is a difference in blood pressure reduction between patients with different *M235T* alleles and explores how this genetic variant contributes to variations in valsartan response compared to other nongenetic factors.

## 2 Materials and Methods

### 2.1 Patients

This study is a cohort study involving 95 individuals, encompassing both genders. The research period was from October 2022 to December 2023. Participants' ages varied from 18 to 73 years. We utilized an upper arm automated blood pressure device (Omron 705IT (HEM‐759‐E)) to measure blood pressure. This device has been validated for accuracy. Blood pressure readings were collected from each participant on a minimum of two occasions before and two occasions after the initiation of valsartan at our outpatient clinics, with a 1‐week interval between each session. Additionally, we compared these clinic measurements with home blood pressure readings to identify and exclude participants showing signs of white coat hypertension. The diagnosis of hypertension adhered to the guidelines outlined by the European Association of Hypertension [11]. Inclusion criteria for the patients were adult Jordanian hypertensive patients with newly diagnosed hypertension who were blindly started the monotherapy 160 mg valsartan treatment with no other co‐morbidities such as diabetes mellitus, chronic kidney disease, coronary artery disease, or thyroid disorders. The researchers ensured that the participants were unrelated by verifying their parents' names and family information. Patients were excluded from this study if the participant was not Jordanian, pregnant, white coat hypertension, or diagnosed with secondary hypertension cases. The participant patients in this study were Arabs, the major ethnic group in Jordan. The Arabic ethnicity was determined in this study through direct questioning and analysis of family names indicative of Arab heritage. Before participating, all individuals provided informed consent. The study's protocol received approval from the ethical committee at the University of Jordan Hospital, adhering to the Helsinki Declaration [12], with the protocol review number 10/2022/24730 on 1/10/2022. Participant demographics were collected from hospital records.

### 2.2 Assessment the Response of Valsartan

The patients' blood pressure was measured twice: first when the nephrologist prescribed 160 mg valsartan, averaging three measurements, and then again after 1 month of using valsartan with averaging three measurements. The response to valsartan was calculated by subtracting the initial systolic or diastolic blood pressure from the blood pressure measured after 1 month of valsartan use. The following equation is used to calculate the response of valsartan response:

– Valsartan's response to the systolic blood pressure = the systolic blood pressure at the time of starting prescribing valsartan to the patient–the systolic pressure blood after 1 month of using valsartan.
– Valsartan's response to the diastolic blood pressure = the diastolic blood pressure at the time of starting prescribing valsartan to the patient–the diastolic blood pressure after 1 month of using valsartan.

### 2.3 Genetic Analysis of AGT M235T Variant

For genetic analysis, around 5 mL of blood samples were collected in EDTA tubes. Genomic DNA was extracted from the leukocytes of these samples using the Wizard Genomic DNA Purification Kit (Promega, Madison, USA), following the manufacturer's protocol. DNA concentration was precisely measured with a Quawell DNA/Protein Analyzer (Sunnyvale, California, USA). The 260/280 ratio of the samples, which indicates the purity, was around 1.8 ± 0.1. Genetic examination focused on amplifying the *AGT gene (T704C, rs699)* through polymerase chain reaction (PCR) (Bio‐Rad, Hercules, CA, USA), employing specific primer pairs, as prescribed previously [13]. Briefly, the reaction mixture included DNA polymerase, dNTPs, MgCl_2_, and buffer in a total volume of 20 μL. The PCR reaction consisted of 35 cycles, each cycle contained the followings: denaturation at 95℃, annealing at 58℃, and extension at 72℃. After that, agarose gel (1.5%) electrophoresis confirmed the PCR products, stained with Redsafe dye (iNTRON, South Korea) for visualization under UV light. The size of the PCR product is 165 base pair after visualization using gel electrophoresis. Then, restriction fragment length polymorphism (RFLP) analysis using *Psyl (Tth 111 I)* enzyme was used for genotyping the *AGT M235T* variant, with results visualized on 2.5% agarose gels under UV light.

**Figure 1:**
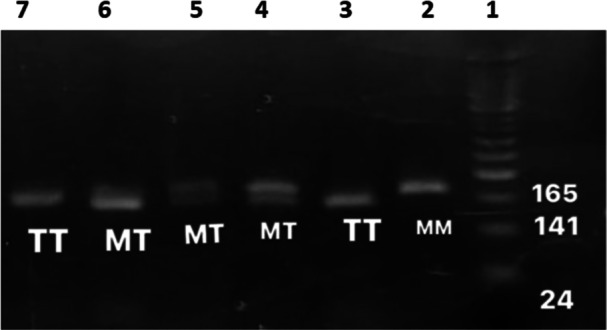
The gel electrophoresis of the *AGT M235T* genotype among the hypertensive patients. The DNA ladder is shown in the lane 1. Lanes 2 indicates the reference *M/M* genotype, lanes 4, 5, and 6 indicate the heterozygous *M/T* genotype, and lanes 3 and 7 indicate the homozygous *T/T* genotype. The size of the PCR products is expressed in base pairs.

The gel electrophoresis of the *AGT M235T* genotype of the hypertensive patients in this study is depicted in Figure 1. The reference (wild) type *M/M* is represented by one band with a size of 165 base pairs. The heterozygous genotype *M/T* is represented by 3 bands, 165, 141, and 24 base pairs. Lastly, the homozygous genotype *T/T* is represented by 2 bands, 141 and 24 base pairs. The genotyping of each volunteer in this study was done twice to confirm the results. To reduce the potential errors in genotyping results, the genetic analysis of each patients was repeated twice. Any discrepancy between the two runs was resolved by performing a third run conducted by a different research assistant to eliminate any potential bias.

### 2.4 Statistical Analysis

The normality of the continuous data was checked used the Shapiro‐Wilk test. The Shapiro‐Wilk test confirmed normality for both SBP and DBP (*p* > 0.05), justifying the use of parametric tests. The comparison between the continuous data of two groups was assessed using *One‐Way t‐student* test, while the *One‐Way ANOVA* test followed by *tukey* post‐hoc test was used to compare the valsartan response among different genotypes. The Pearson correlation and the binary regression (with coding of male = 0, female = 1, nonsmoker = 0, and smoker = 1, *Chi‐square* test) were applied to find the association of the anthropometric parameters of the patients with the valsartan response. SPSS software (version 26, IBM, USA) was used in the statistical analysis of the data. A *p*‐value below 0.05 was considered statistically significant.

## 3 Results

### 3.1 Anthropometric Data

The data in Table 1 show the anthropometric parameters of the participants in this study. The mean age of the patients was 45.82 ± 10 years, with an average height of 168.85 ± 11 cm. The mean body weight stands at 87.94 ± 18 kg. The calculated body mass index (BMI) of the participants averages at 30.82 ± 5.5, placing this sample of hypertensive patients within the obese classification [14]. The sample encompasses both male and female constituents, with 46 male and 49 female participants. Regarding the smoking status of the participants, 28 reported smoking habits, while 67 participants identified themselves as non‐smokers.

**Table 1:** Anthropometric data of the hypertensive patients in this study.

Age	Height	Weight	BMI	Gender	Smoking
**45.82 ± 10**	168.85 ± 11	87.94 ± 18	30.82 ± 5.50	Male: 46 (48%)	Yes 28 (29%)
				Female: 49 (52%)	No 67 (71%)

*Note:* The data are represented in mean ± standard deviation. BMI is the abbreviation of the body mass index.

### 3.2 Response Toward Valsartan

Table 2 illustrates pre and postintervention SBP and DBP measurements, along with the values of the differences in the values of SBP and DBP pre and postone month of valsartan treatment. Before the valsartan administration, the mean SBP was 160.36 ± 14.03 mmHg, while the mean DBP was 98.86 ± 6.02 mmHg. After 1 month of the valsartan treatment, the mean SBP decreased to 124.61 ± 8.85 mmHg, and the mean DBP also decreased to 79 ± 6.48 mmHg. The observed changes, represented as “Δ SBP” and “Δ DBP,” represent the differences between the post‐ and pre‐ valsartan's treatment values, yielding a mean ΔSBP of 35.75 ± 15.19 mmHg, and a ΔDBP of 19.57 ± 8.06 mmHg.

**Table 2:** Values of systolic and diastolic blood pressure pre and postone month of valsartan treatment among a sample of hypertensive patients in Jordan.

	SBP pre	DBP pre	SBP post	DBP post	ΔSBP	ΔDBP
Mean	160.36 ± 14.03	98.86 ± 6.02	124.61 ± 8.85*	79.00 ± 6.48*	35.75 ± 15.19	19.57 ± 8.06
95% CI	157.50–163.22	97.63–100.09	122.81–126.41	77.68–80.32	32.66–38.84	17.93–21.21

*Note:* SBP is the abbreviation of systolic blood pressure and DBP is the abbreviation of the diastolic blood pressure.

* Indicates a statistical significance with a *p* value less than 0.05 using *t*‐test.

### 3.3 Interindividual Variation in the Response of Valsartan

**Figure 2:**
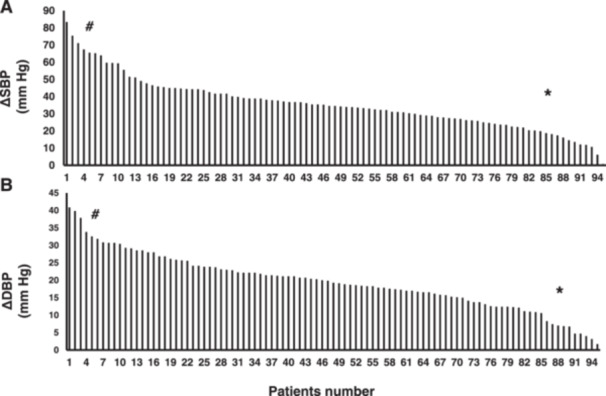
Interindividual variation toward valsartan's response. The assessment of hypertensive patients' response to one month of valsartan treatments was based on the difference in systolic (A) and diastolic (B) blood pressure. In the figures, “#” denotes patients exhibiting a high response, while “*” indicates patients with a low response to valsartan.

Figure 2A,B illustrate the considerable interindividual variability in the response to valsartan among this cohort of Jordanian hypertensive patients. In some patients, SBP was reduced by more than 70 mmHg following valsartan treatment. In contrast, others experienced a reduction in SBP of less than 10 mmHg, as shown in Figure 2A. Similarly, reductions in DBP varied significantly, with some patients experiencing a decrease of 30 mmHg, while others saw reductions of less than 2 mmHg, as depicted in Figure 2B.

### 3.4 AGT M235T Genotype Among the Participants

The Table 3 shows how different *AGT M235T* genotype within this sample of hypertensive patients. There are 27 (28%) individuals with the “reference” genotype, 50 (53%) with heterozygous, and 18 (19%) with the homozygous genotype. The frequency of these *AGT M235T* genotypes was not deviated from the Hardy‐Weinberg equation (*Chi‐square*, *p* > 0.05).

**Table 3:** The frequency of *AGT M235T* genotype among the participants.

*AGT M235T* genotype	Number (%)	95% CI
Reference	27 (28%)	19.41–37.53
Heterozygous	50 (53%)	43–63
Homozygous	18 (19%)	11–27

### 3.5 The Influence of AGT M235T Genotype on Valsartan Response

The results shows that hypertensive patients with the homozygous *AGT M235T* genotype had an average ΔSBP of 31.44 ± 10.75 mmHg, and an average ΔDBP of 16.85 ± 5.6 mmHg which are less significantly (*ANOVA*, *p* < 0.05) than the ΔSBP and ΔDBP values of the reference and heterozygous *AGT M235T* genotypes using both codominant and recessive genotyping model, as illustrated in Table 4.

**Table 4:** Valsartan response among the hypertensive patients in accordance with the *AGT M235T genotype*.

*Genotyping model*	*AGT M235T* genotype	ΔSBP	95% CI	ΔDBP	95% CI
	Reference	39.70 ± 16	36.89–42.51	20.51 ± 8	18.75–22.27
Codominant	Heterozygous	35 ± 15	31.88–38.12	20 ± 8.52	17.72–22.28
	Homozygous	31.44 ± 10.75*	28.58–34.30	16.85 ± 5.63*	14.81–18.89
Recessive	Reference + Heterozygous	37.21 ± 16	34.41–40.01	20 ± 7.13	18.43–21.57
	Homozygous	31.44 ± 10.75*	28.58–34.30	16.85 ± 5.64*	14.81–18.90

*Note:* The response toward valsartan response is indicated by ΔSBP and ΔDBP. ΔSBP is the difference in systolic blood pressure and ΔDBP is the abbreviation of the difference in the diastolic blood pressure pre‐ and post‐valsartan treatment.

* Indicates statistical significance with *p* < 0.05 using *One‐way ANOVA* test for the codominant and *t‐test* for the recessive genotyping model.

### 3.6 The Correlation of Patient's Age, Height, and BMI With the Valsartan Response

**Figure 3:**
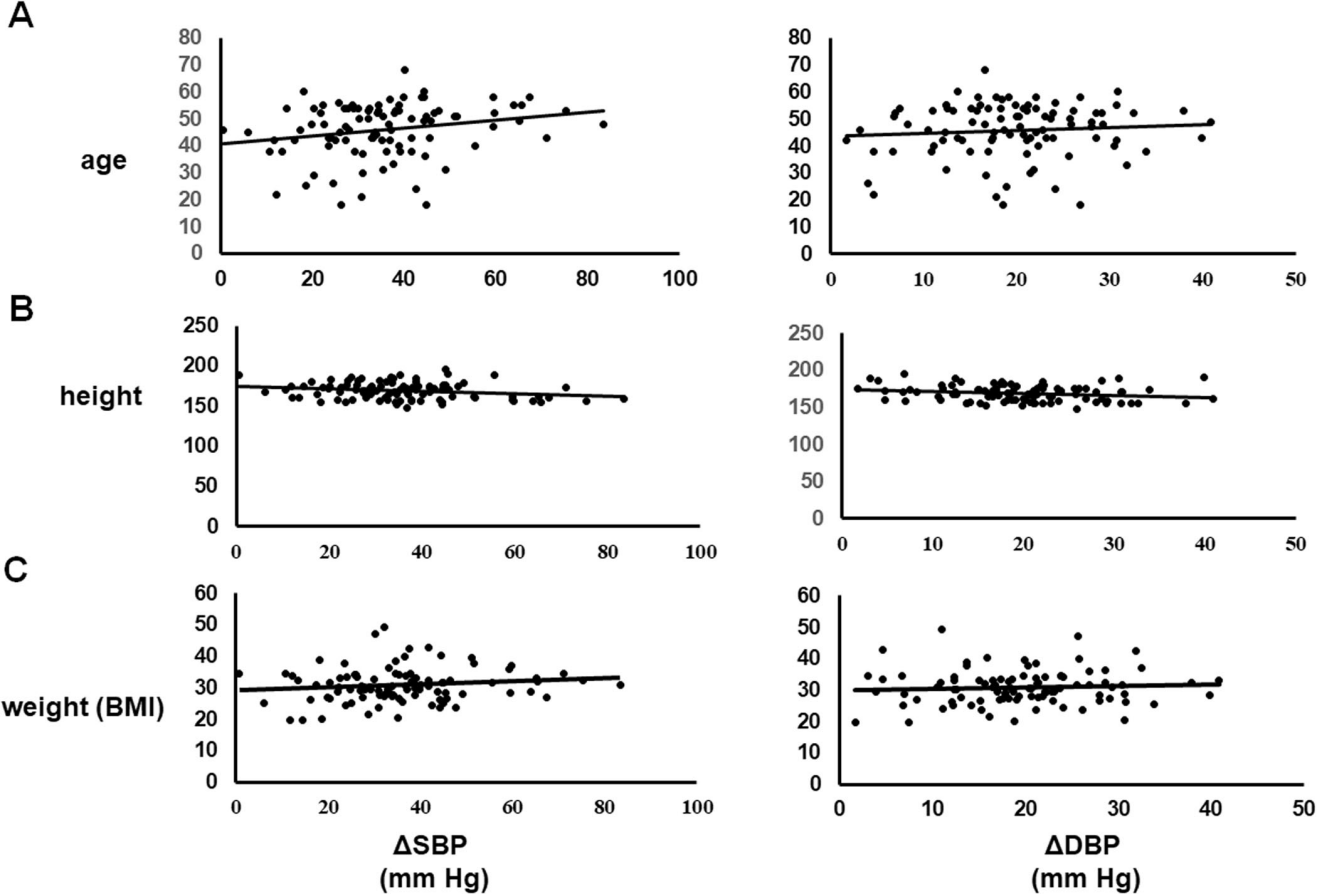
The correlation between the patient's age (A), height (B), and BMI (C) with the valsartan response. The response toward valsartan response is indicated by ΔSBP and ΔDBP. ΔSBP is the difference in systolic blood pressure and ΔDBP is the abbreviation of the difference in the diastolic blood pressure pre‐ and post‐valsartan treatment.

Figure 3 shows the correlation of valsartan response with the age, height, and BMI of the participant patients, while Table 5 shows the statistical analysis of these correlations. Age shows a positive significant correlation with ΔSBP (*R* = 0.224, *p* = 0.03), although the effect on ΔDBP is not statistically significant (*R* = 0.081, *p* = 0.44). Height is negatively correlated with both ΔSBP (*R* = −0.231, *p* = 0.02) and ΔDBP (*R* = −0.193, *p* = 0.04). BMI shows a weak and nonsignificant correlation with changes in blood pressure following valsartan treatment, with *R* scores of 0.135 (*p* = 0.195) for ΔSBP and 0.057 (*p* = 0.58) for ΔDBP.

**Table 5:** Correlation of age, height, BMI with the valsartan response.

	R score	ΔSBP		R score	ΔDBP	
		95%CI	*p* value		95% CI	*p* value
Age	0.22	0.02 to 0.42	0.03*	0.08	−0.12 to 0.28	0.44
Height	−0.23	−0.43 to −0.03	0.02*	−0.19	−0.39 to 0.01	0.04*
BMI	0.14	−0.06 to 0.34	0.20	0.06	−0.14 to 0.26	0.58

* Indicates statistical significance (*p* < 0.05). The response toward valsartan response is indicated by ΔSBP and ΔDBP. ΔSBP is the difference in systolic blood pressure and ΔDBP is the abbreviation of the difference in the diastolic blood pressure pre‐ and post‐valsartan treatment.

### 3.7 The Association of Patient's Sex and Smoking Status With the Response Toward Valsartan

Table 6 presents the association between sex, smoking status, and the response to valsartan, with reported regression coefficients for each variable. For sex, the coefficient is 5.2 for ΔSBP and 2.7 for ΔDBP, suggesting a positive association between being female and an increase in the response to valsartan. However, this association is statistically significant (*p* = 0.006) for the systolic response but not statistically significant (*p* = 0.12) for the diastolic response to valsartan.

**Table 6:** Binary regression of the sex and smoking status with the valsartan response.

	*Regression coefficient, p* value	
**Parameter**	ΔSBP	ΔDBP
**Sex**	5.21, 0.01*	2.73, 0.12
**Smoking**	0.93, 0.87	1.74, 0.61

* Indicates statistical significance (*p* < 0.05, *Chi‐square*). The response toward valsartan response is indicated by ΔSBP and ΔDBP. ΔSBP is the difference in systolic blood pressure and ΔDBP is the abbreviation of the difference in the diastolic blood pressure pre‐ and post‐valsartan treatment.

Regarding the smoking status of the patients, the statistical analysis showed that there is no statistically significant association (*p* > 0.05) between smoking and the valsartan response, as illustrated in Table 6.

## 4 Discussion

There is interindividual and interethnic variation in the response toward antihypertensive drugs [14]. We noticed in the University of Jordan hospital that there is a wide variation in the response of valsartan among the hypertensive patients attending the university's hospital. However, factors influencing the response toward valsartan are still unknown. In this study, we investigated the association of genetic, particularly *AGT M235T* genetic variant, and other anthropometric parameters with the response of valsartan. We found in this study that hypertensive patients with the homozygous mutant *AGT M235T* genotype had a significantly less response toward valsartan than those with the heterozygous and reference *AGT M235T* genotype. In addition, we found that older, shorter, and female patients have a tendency to respond more toward valsartan than younger, taller, and male hypertensive patients. These results can help us in understanding more about the valsartan response and optimizing its pharmacotherapy among hypertensive patients in Jordan. However, these data are still preliminary, and we need further evidence using a larger number of patient samples and multicenter investigations.

Valsartan was chosen as the drug to study in this investigation since it is the most commonly prescribed drug in the University of Jordan hospital. Other ARB drugs are less prescribed in the University of Jordan hospital. Although other ARBs have a similar mechanism of action in the management of cardiovascular diseases, the findings of this study are applied to valsartan and still cannot be applied to other ARBs due to differences in the chemical structure and hence the pharmacokinetics of the ARBs [15].

This study introduces some novel ideas to high blood pressure research. First, it investigates how the genetic variant *AGT M235T* might influence the effectiveness of valsartan response. Additionally, it explores whether factors like age, height, and weight could impact how well valsartan works for patients. This study provides new insights into the potential influence of these factors on treatment response among hypertensive patients. Lastly, the focus on the Jordanian population and practices at the University of Jordan hospital, the major educational hospital in Jordan, also contributes unique insights to the field.

We previously found that the BMI is associated with an increased risk of essential hypertension among Jordanians [13]. Interestingly, in this study, we found that most of the hypertensive participants were obese, as indicated by their BMI values. This finding confirms that obesity is associated with essential hypertension in Jordanians.

Genetic studies have linked *AGT* gene variants, including the *M235T* genetic variant, to cardiovascular diseases [16], making it a pertinent target for investigation in the context of ARBs response. We recently found that the *AGT M235T* genotype was not associated with essential hypertension in Jordan [13]. In addition, we found that there is no statistical significance in these *AGT M235T* genotype frequencies in this study with what was reported previously about the frequency of *AGT M235T* among Jordanian hypertensive patients [13].

However, in this study, we found that the mutant allele of the *AGT M235T* genotype, in a recessive genotyping model, decreases the response significantly toward valsartan. Substitution of methionine to threonine at amino acid number 235 of the angiotensinogen protein increases the polarity of the *AGT* protein and affects its stability and hence its processing in the activated RAS. Since the response toward valsartan is correlated with the activation of RAS, the *AGT M235T* variant may reduce the activation of RAS and hence reduce the response toward valsartan [17, 18]. However, further molecular mechanisms are needed to understand the mechanism of the *AGT M235T* genetic variation on the valsartan response.

Identifying hypertensive patients who have the homozygous mutant *AGT M235T* genotype might help in predicting a lower response to valsartan. This means that genetic testing for this variant could be considered before giving valsartan, especially for patients who do not have good blood pressure control. In the future, clinical guidelines might include *AGT* genotyping, with other molecular biomarkers [19], as part of the process for choosing antihypertensive treatment, which could help move toward more personalized treatment for hypertension [20].

Correlation analyses highlighted age and height as influential factors, indicating that older individuals experienced a more pronounced reduction in SBP, while taller individuals exhibited a lesser response to valsartan in both systolic and diastolic measures.

The impact of age on the valsartan response aligns with what was reported in the previous literature, suggesting age‐related variations in the drug's pharmacokinetic parameters, such as reduction in metabolism and excretion, and hence alteration in the drug's efficacy [21]. While the association between age and the valsartan response was statistically significant for systolic blood pressure, the lack of statistical significance for diastolic blood pressure warrants further exploration.

In contrast, the negative correlation between height and the valsartan response implies that taller individuals may experience a diminished reduction in blood pressure with valsartan therapy. The reasons behind this association are still unknown, but it might be due to the fact that the patient's height affects cardiovascular parameters and hence may affect the drug's response [22].

Binary regression analysis of sex and smoking status with valsartan response provided additional insights. The significantly positive association of female sex with increased ΔSBP aligns with existing studies suggesting gender‐specific variations in antihypertensive drug responses [23]. However, the lack of a significant association between smoking status and valsartan response suggests that smoking may not be a major factor influencing the efficacy of valsartan in this population.

The notable strengths of our study include its exploration of genetic and demographic factors on the valsartan response. However, certain limitations should be acknowledged. The absence of long‐term follow‐up data might limit our understanding of sustained treatment responses. However, Lasserson et al. (2011) found that antihypertensive drugs, including valsartan, reach 50% of their maximum effect within 1 week. This means that most of the drug's response happens early in treatment [24]. Additionally, the study's focus on the *AGT M235T* variant, while informative, does not encompass the entirety of genetic influences on valsartan response, warranting further genetic exploration. In addition, this study did not evaluate the patient's adherence to valsartan treatment and diet that can influence on the pharmacokinetics of valsartan, which can one a factor of variability in the response. Lastly, it's important to note that the sample size in this study is relatively small, suggesting that it can be considered a pilot study. Although, we followed a restricted inclusion criteria that the patient has only essential hypertension with no other comorbidities, including diabetes, to minimize factors affecting the response to valsartan. We made our best effort to recruit as many patients as possible over 1 year. Further clinical investigations with larger sample sizes and multi‐center approaches are necessary to validate the findings of this study.

## 5 Conclusions

In conclusion, the *AGT M235T* genotype, along with patient's sex, height, and age, are factors that influence the valsartan response. These findings can aid in understanding the interindividual variation in the response to valsartan and underscore the potential for personalized hypertension management. Further multicenter studies with larger sample sizes are needed to confirm the findings of this study.

## Author Contributions

**Hussein Alhawari:** investigation, methodology, writing – original draft. **Yazun Jarrar:** conceptualization, data curation, investigation, methodology, supervision, writing – original draft, writing – review and editing. **Malek Zihlif:** supervision. **Ayman Wahbeh:** data curation. **Sameeha Alshelleh:** data curation. **Khaled Ojjoh:** data curation. **Dalia Abdelrazaq:** data curation. **Hussam Alhawari:** data curation.

## Ethics Statement

The study's protocol received approval from the ethical committee at the University of Jordan Hospital, adhering to the Helsinki Declaration, with the protocol review number 10/2022/24730 on 1/10/2022. Participant demographics were collected from hospital records.

## Conflicts of Interest

The authors declare no conflicts of interest.

## Transparency Statement

The lead author Yazun Jarrar affirms that this manuscript is an honest, accurate, and transparent account of the study being reported; that no important aspects of the study have been omitted; and that any discrepancies from the study as planned (and, if relevant, registered) have been explained.

## Data Availability

The authors confirm that the data supporting the findings of this study are available within the article.
